# Comparative study of Claude 3.5-Sonnet and human physicians in generating discharge summaries for patients with renal insufficiency: assessment of efficiency, accuracy, and quality

**DOI:** 10.3389/fdgth.2024.1456911

**Published:** 2024-12-05

**Authors:** Haijiao Jin, Jinglu Guo, Qisheng Lin, Shaun Wu, Weiguo Hu, Xiaoyang Li

**Affiliations:** ^1^ Department of Nephrology, Ren Ji Hospital, Shanghai Jiao Tong University School of Medicine, Shanghai, China; ^2^ Department of Nephrology, Ningbo Hangzhou Bay Hospital, Zhejiang, China; ^3^ Molecular Cell Lab for Kidney Disease, Shanghai, China; ^4^ Shanghai Peritoneal Dialysis Research Center, Shanghai, China; ^5^ Uremia Diagnosis and Treatment Center, Shanghai Jiao Tong University School of Medicine, Shanghai, China; ^6^ WORK Medical Technology Group LTD., Hangzhou, China; ^7^ Department of Medical Education, Ruijin Hospital Affiliated to Shanghai Jiao Tong University School of Medicine, Shanghai, China

**Keywords:** artificial intelligence, discharge summaries, renal insufficiency, claude 3.5-Sonnet, medical documentation

## Abstract

**Background:**

The rapid development of artificial intelligence (AI) has shown great potential in medical document generation. This study aims to evaluate the performance of Claude 3.5-Sonnet, an advanced AI model, in generating discharge summaries for patients with renal insufficiency, compared to human physicians.

**Methods:**

A prospective, comparative study was conducted involving 100 patients (50 with acute kidney injury and 50 with chronic kidney disease) from the nephrology department of Ningbo Hangzhou Bay Hospital between January and June 2024. Discharge summaries were independently generated by Claude 3.5-Sonnet and human physicians. The main evaluation indicators included accuracy, generation time, and overall quality.

**Results:**

Claude 3.5-Sonnet demonstrated comparable accuracy to human physicians in generating discharge summaries for both AKI (90 vs. 92 points, *p* > 0.05) and CKD patients (88 vs. 90 points, *p* > 0.05). The AI model significantly outperformed human physicians in terms of efficiency, requiring only about 30 s to generate a summary compared to over 15 min for physicians (*p* < 0.001). The overall quality scores showed no significant difference between AI-generated and physician-written summaries for both AKI (26 vs. 27 points, *p* > 0.05) and CKD patients (25 vs. 26 points, *p* > 0.05).

**Conclusion:**

Claude 3.5-Sonnet demonstrates high efficiency and reliability in generating discharge summaries for patients with renal insufficiency, with accuracy and quality comparable to those of human physicians. These findings suggest that AI has significant potential to improve the efficiency of medical documentation, though further research is needed to optimize its integration into clinical practice and address ethical and privacy concerns.

## Introduction

The rapid development of Artificial Intelligence (AI) technology is profoundly transforming various aspects of the healthcare system, with the field of medical document generation attracting particular attention. Advanced language models based on Natural Language Processing (NLP), such as ChatGPT and Claude, have demonstrated exceptional performance in generating various types of medical documents, garnering widespread interest from both academia and industry (1). These AI models not only show potential for improving efficiency but also promise to address long-standing challenges in healthcare document management (2, 3).

Medical documentation plays a central role in modern healthcare systems, with its importance manifested in multiple areas: quality of patient care, operational efficiency of medical institutions, and legal compliance. Accurate and timely medical records directly impact patient diagnosis and treatment outcomes, while also being crucial for improving service efficiency and meeting legal requirements in healthcare institutions. However, traditional manual document generation methods face issues of low efficiency and error-proneness. Studies have shown that human-written discharge summaries often contain errors or omissions, with rates ranging from 13% to 40% depending on the type of information (4, 5). To address these issues, several organizations have published guidelines for discharge summary content and structure, such as those by the Joint Commission and the American College of Physicians (6, 7). Against this backdrop, the introduction of AI technology offers new possibilities for addressing these long-standing challenges while adhering to established standards.

Medical documentation plays a central role in modern healthcare systems, with its importance manifested in multiple areas: quality of patient care, operational efficiency of medical institutions, and legal compliance. Accurate and timely medical records directly impact patient diagnosis and treatment outcomes, while also being crucial for improving service efficiency and meeting legal requirements in healthcare institutions. However, traditional manual document generation methods face issues of low efficiency and error-proneness. Against this backdrop, the introduction of AI technology offers new possibilities for addressing these long-standing challenges.

Recent studies have begun to explore the potential applications of AI in medical document generation, yielding encouraging results. Baker et al. (8) demonstrated that ChatGPT can effectively generate medical history records and discharge summaries, not only reducing physicians' workload but also improving document consistency and standardization. Further studies (9–11) confirmed that AI can provide high-quality, clinically meaningful text in generating complex medical documents such as surgical records, progress notes, and discharge summaries. These research findings have laid a solid foundation for the widespread application of AI in medical document generation.

However, AI-generated medical documents also face some challenges. Hofmann et al. (12) pointed out that AI still needs further optimization in the use of certain specialized medical terminology and handling of specific details. More concerningly (8), found that AI-generated texts sometimes contain erroneous information, which could potentially negatively impact the quality of patient care. These findings emphasize the importance of maintaining caution and critical thinking when applying AI technology to medical document generation.

On June 21, 2024, Anthropic released Claude 3.5 Sonnet (13), the first version in the Claude 3.5 series of models. According to official statements, this model outperforms competing models such as GPT-4 (14), Gemini 1.5 (15), and Llama-400b (16), as well as its predecessor, Claude 3 Opus, in various evaluations, while also offering faster response times. This breakthrough provides the latest and most advanced tool for researching AI applications in medical document generation.

In the field of nephrology, particularly for complex diseases such as Acute Kidney Injury (AKI) and Chronic Kidney Disease (CKD), accurate medical documentation is crucial for long-term patient management and prognosis evaluation. However, research on AI applications in this specific area remains relatively limited. In light of this, our study aims to evaluate the performance of Claude 3.5 Sonnet, currently the best-performing model overall, in generating discharge summaries for kidney disease patients (especially those with AKI and CKD), and to comprehensively compare it with human physicians.

Through a multi-dimensional comparative analysis, this study aims to comprehensively assess the potential and limitations of AI technology in clinical practice, with a focus on the generation of medical documents. Specifically, we evaluate the performance of Claude 3.5-Sonnet in producing discharge summaries for patients with renal insufficiency, comparing its accuracy, efficiency, and quality to those generated by human physicians. Our findings will not only provide empirical evidence supporting the application of AI in medical documentation but also offer crucial insights for future AI model optimization and development. Additionally, we explore the ethical and legal implications of using AI in medical document generation, particularly regarding privacy concerns and HIPAA compliance. The novel contributions of this study include the first comprehensive evaluation of Claude 3.5-Sonnet's performance in this context, an in-depth analysis of its ability to handle complex medical terminology and details, and a discussion of the ethical considerations surrounding AI-assisted medical documentation. Ultimately, this study serves as a valuable reference for the responsible integration of AI technologies in the medical field.

## Methods

### Research design

This study employs a controlled research design aimed at comparing the performance of Claude 3.5-Sonnet with human physicians in generating discharge summaries for patients with acute kidney injury (AKI) and chronic kidney disease (CKD). The study includes 100 patients with renal insufficiency, comprising 50 AKI and 50 CKD cases. Discharge summaries for each patient are independently generated by both Claude 3.5-Sonnet and human physicians. The main evaluation indicators include document accuracy, generation time, and overall quality.

### Study subjects

The study subjects consist of 50 consecutive cases of acute renal insufficiency and 50 cases of chronic renal insufficiency treated at the nephrology department of Ningbo Hangzhou Bay Hospital from January to June 2024. Inclusion criteria: (1) age ≥18 years; (2) confirmed diagnosis of AKI or CKD; (3) hospital stay ≥7 days. Exclusion criteria: (1) concurrent major organ failure; (2) cognitive impairment; (3) refusal to participate in the study. All patients signed informed consent forms.

#### Dataset and preprocessing

The dataset used in this study includes patient information, admission records, daily progress notes, medical orders, and discharge treatment recommendations. To ensure the data input into the Claude 3.5-Sonnet model meets the model's requirements, the following preprocessing steps were undertaken:

1. Data Anonymization: In compliance with privacy regulations (such as HIPAA compliance), all patient data were anonymized. Sensitive information such as names, identification numbers, and hospital admission numbers were removed. This anonymization process was strictly followed throughout the data handling and analysis to protect patient privacy.
2. Text Standardization: Prior to inputting the patient records into Claude 3.5-Sonnet, the text was standardized. This involved correcting spelling errors, unifying medical terminology, removing unnecessary repetitive information, and ensuring consistency and readability of the data. This preprocessing step aimed to improve Claude 3.5's ability to accurately interpret the input data and generate coherent discharge summaries.
3. Format Adjustment: We adjusted the format of the data according to Claude 3.5's input requirements, ensuring that each section of the content (e.g., progress notes, medical orders, treatment recommendations) was clearly segmented. This enabled the model to better understand and generate the corresponding discharge summaries accurately.

### Discharge summary generation process

#### Claude 3.5-Sonnet generation process

Research assistants upload patient information, admission records, daily progress notes, medical orders, and discharge treatment recommendations into the system. Using the prompt “Please generate a discharge summary based on the provided materials,” Claude 3.5-Sonnet automatically generates discharge summaries. The total time from data input to summary generation is recorded for the entire process. Additionally, we implemented standardized medical history templates and a scoring rubric for discharge summaries as the basis for retrieval-augmented generation (RAG).

#### Human physician generation process

Five nephrologists, each with over five years of clinical experience, are randomly assigned writing tasks. They independently write discharge summaries based on the same patient information, and the time from starting to write to completing the final draft is recorded.

### Evaluation indicators and methods

#### Accuracy assessment

The evaluation is conducted by an expert review panel consisting of three chief physicians in nephrology. The scoring criteria include diagnostic accuracy (0–40 points), treatment process description (0–30 points), and discharge recommendation reasonability (0–30 points). The review panel was blinded to the source of the discharge summaries (AI-generated or human-written).

Each expert independently evaluated all discharge summaries in multiple sessions over a two-week period to prevent fatigue and maintain consistency. The experts did not interact with each other during the evaluation process. The evaluation was based on a scoring rubric developed for this study, which incorporated elements from existing practice standards and guidelines for discharge summary preparation, including those from the Joint Commission and the American College of Physicians (6, 7). The full scoring rubric is provided in Appendix A.

#### Time efficiency assessment

For Claude 3.5-Sonnet, the time from data input to summary generation is recorded. For human physicians, the time from starting to write to completing the final draft is recorded.

#### Overall quality assessment

The evaluation dimensions include completeness (0–10 points), logical coherence (0–10 points), and readability (0–10 points). The review panel independently evaluated all discharge summaries, with each expert scoring each dimension, and the final score was calculated as the average of the three experts' scores.

### Statistical analysis

Data analysis was performed using SPSS 26.0 software. Continuous variables are expressed as mean ± standard deviation, and categorical variables as frequency and percentage. Paired sample *t*-tests were used to compare differences in accuracy and overall quality scores between Claude 3.5-Sonnet and human physicians. Independent sample *t*-tests were used to compare differences in generation time between the two groups. Interrater reliability was assessed using the intraclass correlation coefficient (ICC). All statistical tests were two-sided, with *P* < 0.05 considered statistically significant.

We performed a power analysis to ensure the study could detect clinically meaningful effect sizes. Based on the sample size and the expected differences between group means, we set Cohen's *d* = 0.2 as the minimum detectable effect size. To further clarify the effect size analysis, we used the pooled standard deviation to calculate the effect size and reported Cohen's *d* as a measure of the difference between the two groups.

Prior to conducting the statistical tests, we assessed the normality of the data distribution using the Shapiro–Wilk test and visual inspection of Q–Q plots. In cases where the normality assumption was violated, we used non-parametric alternatives (Wilcoxon signed-rank test for paired comparisons and Mann–Whitney *U* test for independent comparisons).

## Results

Our study compared the performance of Claude 3.5-Sonnet with human physicians in generating discharge summaries for patients with acute kidney injury (AKI) and chronic kidney disease (CKD). We evaluated three key aspects: accuracy, overall quality, and time efficiency. The interrater reliability for the expert panel's evaluations was high, with an intraclass correlation coefficient (ICC) of 0.92 (95% CI: 0.89–0.95), indicating excellent agreement among raters.

### Accuracy

**Figure 1:**
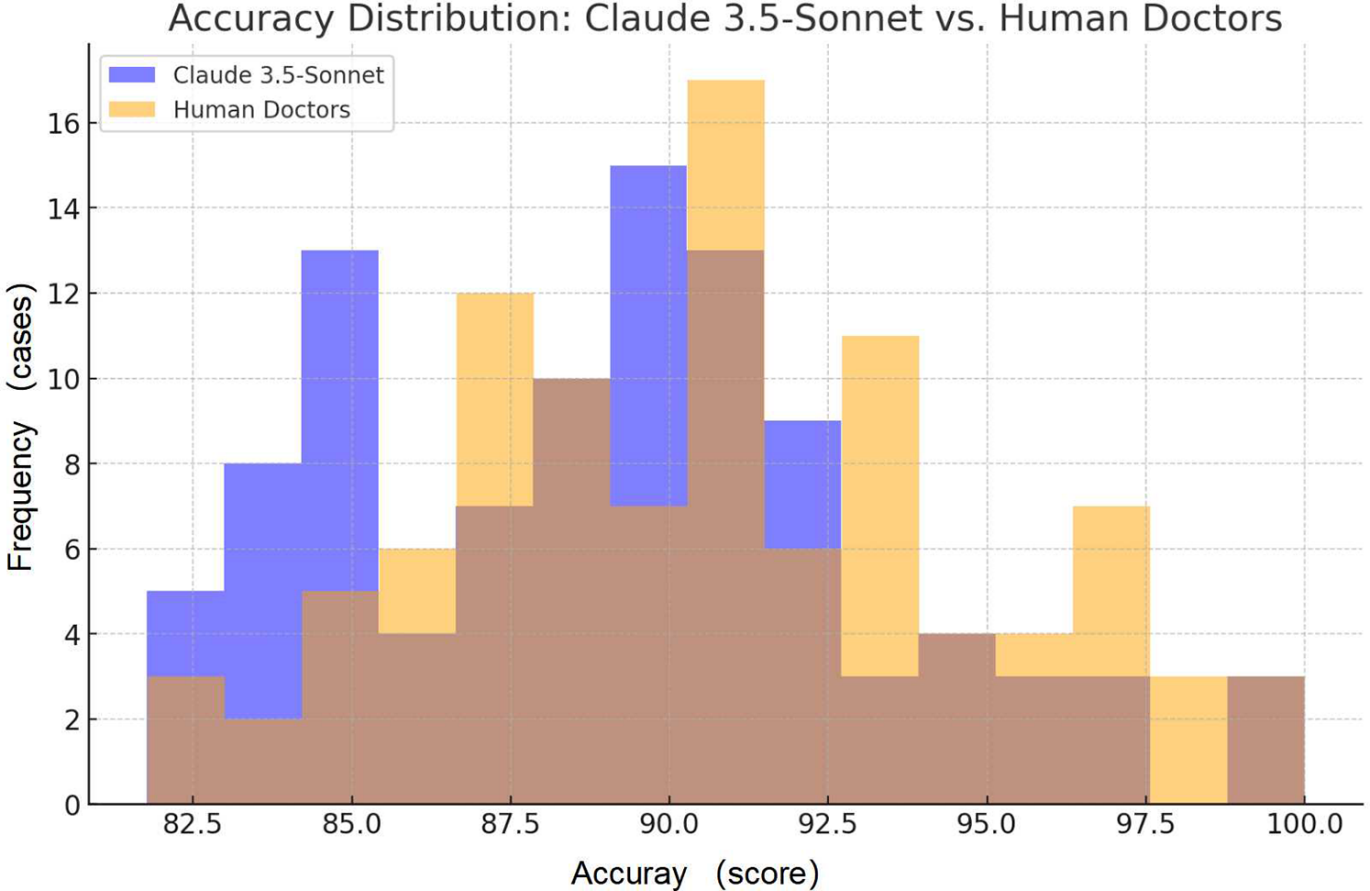
Comparison of accuracy scores: the frequency distribution of accuracy for Claude 3.5-Sonnet and human doctors.

The accuracy of discharge summaries was assessed on a 100-point scale. Our findings reveal comparable performance between Claude 3.5-Sonnet and human physicians (Table 1, Figure 1). Based on the effect size analysis, the calculated Cohen's *d* was −0.129, indicating that the difference between the two groups is minimal and not clinically meaningful.

**Table 1:** Comparison of accuracy scores.

Group	Overall	AKI	CKD
Claude 3.5-Sonnet	90.32 ± 3.75	90.24 ± 3.75	90.40 ± 3.78
Human Physicians	90.81 ± 3.86	90.72 ± 3.61	90.90 ± 4.13
*P*-value	0.391	0.515	0.571

### Overall quality

**Figure 2:**
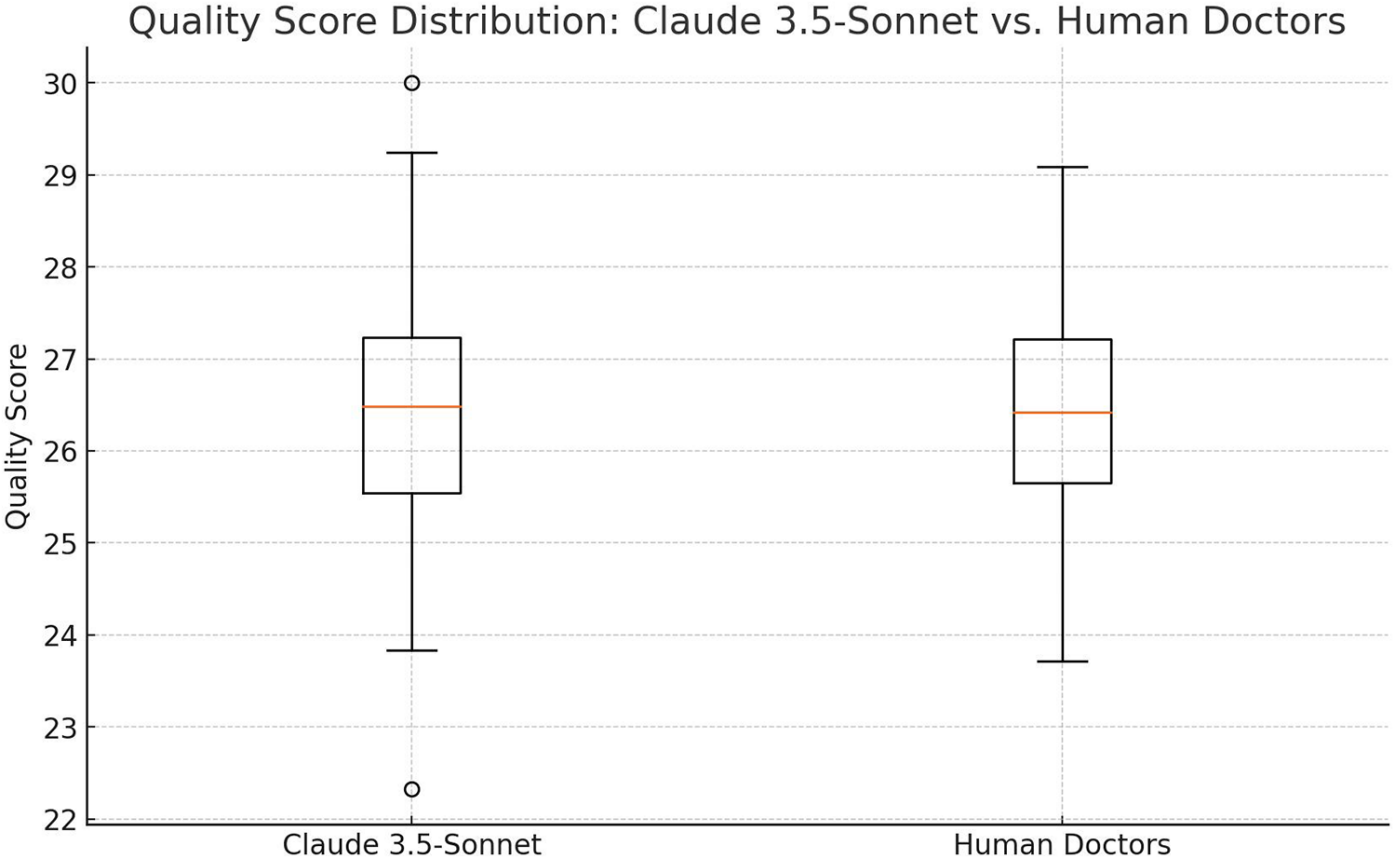
Comparison of quality scores boxplot: the distribution of quality scores between Claude 3.5-Sonnet and human doctors, showing the median and interquartile range for each.

The overall quality of discharge summaries was evaluated on a 30-point scale, considering factors such as completeness, logical coherence, and readability. Our analysis shows similar performance between Claude 3.5-Sonnet and human physicians (Table 2, Figure 2).

**Table 2:** Comparison of overall quality scores.

Group	Overall	AKI	CKD
Claude 3.5-Sonnet	26.43 ± 1.31	26.53 ± 1.28	26.33 ± 1.35
Human Physicians	26.49 ± 1.10	26.40 ± 0.96	26.57 ± 1.23
*P*-value	0.710	0.585	0.392

### Time efficiency

**Figure 3:**
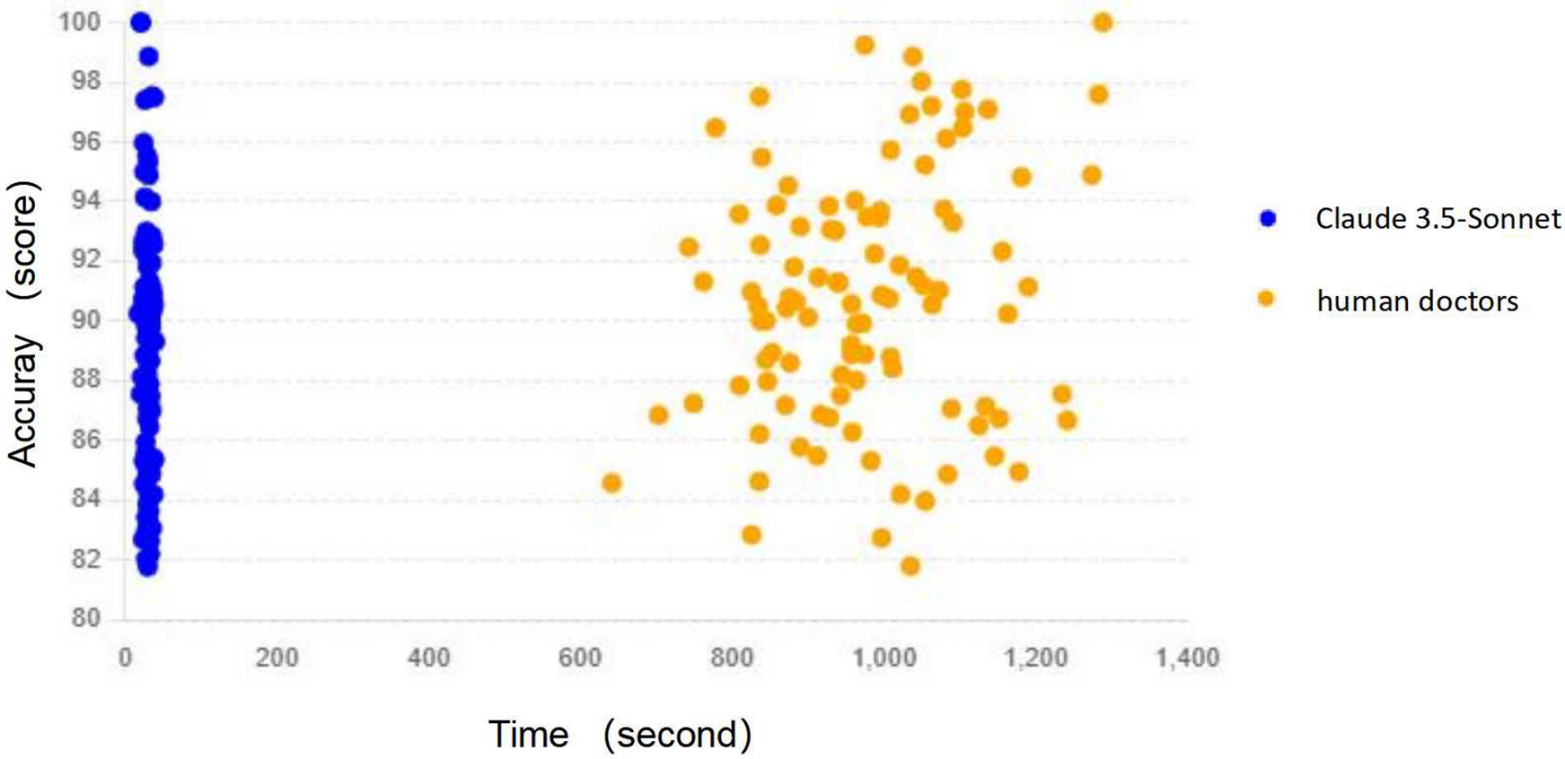
Time vs. accuracy scatter plot: the relationship between time and accuracy for both Claude 3.5-Sonnet and human doctors, helping to understand the correlation between these variables.

The most striking difference between Claude 3.5-Sonnet and human physicians was observed in the time required to generate discharge summaries (Table 3, Figure 3):

**Table 3:** Comparison of generation time.

Group	Overall	AKI	CKD
Claude 3.5-Sonnet	29.48 ± 4.59 s	29.40 ± 4.64 s	29.56 ± 4.59 s
Human Physicians	16.3 ± 2.21 min	18.0 ± 1.44 min	14.5 ± 1.20 min
*P*-value	<0.001	<0.001	<0.001

These results highlight the remarkable time efficiency of Claude 3.5-Sonnet, which generated discharge summaries approximately 33 times faster than human physicians, while maintaining comparable accuracy and quality.

## Discussion

In recent years, the application of artificial intelligence (AI) in medical document generation has garnered widespread attention. Several studies have demonstrated that advanced language models based on natural language processing (NLP) exhibit significant potential in generating medical documents. For example, Baker et al. (8) showed that ChatGPT effectively generates medical history records and discharge summaries, not only reducing physicians' workload but also improving document consistency and standardization. Similarly, research by Schwarz et al. (5) found that AI can produce high-quality, clinically meaningful documents such as surgical reports and progress notes. However, these studies also highlighted that AI-generated medical documents still require optimization in the use of specific medical terminology and in handling details.

Despite the potential, the practical application of AI in medical documentation faces several challenges. Hofmann et al. (12) pointed out that AI-generated texts sometimes contain erroneous information, which could negatively impact patient care. Therefore, caution and critical thinking are essential when applying AI to generate medical documents. Notably, in 2024, Anthropic released the Claude 3.5-Sonnet model, which has surpassed its predecessor Claude 3 Opus and other competing models such as GPT-4 and Gemini 1.5 in various evaluations (17).

Although previous studies have laid a strong foundation for the application of AI in medical document generation, research specifically focusing on its use in nephrology remains limited. Given the complexity of managing patients with acute kidney injury (AKI) and chronic kidney disease (CKD), where accurate medical documentation is critical for long-term management and prognosis evaluation, there is a clear need for further exploration. This study is the first to systematically assess the potential of AI, specifically Claude 3.5-Sonnet, in nephrology by comparing its ability to generate discharge summaries with those written by human physicians. The findings highlight the significant potential of AI in medical documentation while also identifying areas that require further investigation to enhance its application in clinical practice.

### Time efficiency

A significant finding of the study is the exceptional time efficiency of Claude 3.5-Sonnet in generating discharge summaries. The AI model takes an average of only about 30 s to complete a discharge summary, while human physicians require over 15 min on average. This improvement in efficiency could have profound implications for clinical practice. In the current context of strained medical resources and heavy workloads for doctors, utilizing AI-assisted tools can significantly reduce the time required for paperwork, allowing medical staff to devote more energy to direct patient care (18). However, we also need to consider whether this high efficiency might affect doctors' in-depth thinking about patients' conditions and personalized treatment. Future research could explore how to improve efficiency while ensuring the quality and personalization of medical decision-making (19).

### Accuracy and quality although

Claude 3.5-Sonnet scored slightly lower than human physicians in terms of accuracy, though the difference was not statistically significant (*p* > 0.05). Based on the effect size analysis (Cohen's *d* = −0.129), the difference between the two is minimal and unlikely to have a meaningful impact in clinical practice. This suggests that Claude 3.5-Sonnet performs comparably to human physicians in terms of accuracy. This finding is encouraging, indicating that AI models are now capable of generating high-quality medical documents comparable to those produced by human physicians. Particularly noteworthy is that in terms of overall quality scores, Claude 3.5-Sonnet's performance was nearly on par with that of human physicians (20). This suggests that AI-generated documents have achieved a high level in terms of completeness, logical coherence, and readability (21).

However, we must also recognize that the accuracy and quality of medical documents directly relate to patient safety and treatment effectiveness. Although statistical results show no significant differences between AI and human physicians, any subtle differences could have important implications in clinical practice. Therefore, future research should analyze the nature and potential impact of these minor differences more deeply and explore how to further improve the accuracy of AI models (22).

### Clinical application prospects

The results of this study provide strong support for the application of AI in the field of medical document generation. The high efficiency and reliability demonstrated by Claude 3.5-Sonnet suggest that it has the potential to become a powerful tool in medical workflows. By reducing the paperwork burden on doctors, AI can help alleviate pressure on the medical system and improve overall efficiency (23). Moreover, standardized documents generated by AI may help reduce human errors and improve the consistency and comparability of medical records (24).

However, we also need to carefully consider the role of AI in medical practice. AI should be viewed as an assistive tool rather than a complete replacement for human physicians' decision-making and judgment. Future research should explore how to best integrate AI into existing medical workflows and how to train medical staff to effectively use these tools (25).

#### Ethical considerations

In this study, we took significant measures to ensure compliance with ethical standards, particularly regarding patient privacy and data security. All patient data used in the study were fully anonymized before being input into the Claude 3.5-Sonnet model, thereby eliminating any personally identifiable information (PII) from the dataset. This anonymization process ensures adherence to the Health Insurance Portability and Accountability Act (HIPAA) regulations, safeguarding patient confidentiality throughout the research. Additionally, we employed strict data handling protocols to protect against unauthorized access and breaches, ensuring that all information was securely processed and stored. The ethical implications of using AI in medical documentation, especially concerning privacy and the responsible use of patient data, were thoroughly considered, and we believe our approach aligns with the highest standards of ethical research practice.

### Limitations and future research directions

Although this study has made important findings, it also has some limitations. First, the sample size is relatively limited (100 kidney disease patients), which may affect the generalizability of the results. Future studies should expand the sample size and include patients with different types of diseases and medical specialties to verify the applicability of Claude 3.5-Sonnet in broader medical fields (26).

Second, this study only focused on the generation of discharge summaries, while medical documents also include admission records, surgical reports, consultation records, and other types. Future research could explore AI's performance in generating these different types of documents to comprehensively assess its potential in the field of medical documentation (27).

Thirdly, a primary limitation of this study is the binary comparison between LLM-generated and physician-written EMRs, omitting a potentially insightful third group combining LLM output with physician input. While our results demonstrate that LLM-generated EMRs approach the quality of those written by physicians, the study does not explore the potential synergies between AI and human expertise. Combining LLM output with physician input could potentially balance both quality and efficiency, leveraging the speed of AI and the nuanced understanding of healthcare professionals. Future research should address this by implementing a three-group design: LLM alone, physician alone, and LLM with physician input. This approach would provide a more comprehensive understanding of how LLMs can best complement human expertise in clinical documentation, potentially leading to optimized workflows and improved EMR quality. It is noteworthy that while preprocessing steps are crucial for ensuring the quality of input to Claude 3.5, they were not included in the efficiency evaluation. This omission may lead to some bias in the estimation of Claude's time efficiency. This oversight highlights the importance of considering all relevant processes and steps when assessing the overall performance of AI systems.

Furthermore, although Claude 3.5-Sonnet performed well in many aspects, there is still room for improvement in the use of medical terminology and handling of certain details. This suggests that we need to further optimize AI models, possibly by increasing domain-specific training data or improving algorithms to enhance their performance in professional medical contexts (28). For example, using retrieval-augmented models and chain-of-thought techniques to provide personalized, evidence-based recommendations could help patients better understand their treatment options (29).

Importantly, we did not fully account for the variability in physician experience, which could affect the comparison of EMR quality. Potential biases in the LLM training data may lead to skewed or incomplete representations in the generated EMRs, particularly for underrepresented patient populations or rare medical conditions. Moreover, the technical limitations of LLMs in understanding complex medical contexts may impact their ability to handle situations requiring nuanced interpretation and context-dependent decision-making. Our study also did not explore the potential synergies between LLM output and physician input, which could provide a more balanced approach in terms of efficiency and accuracy.

Lastly, this study primarily focused on objective indicators such as efficiency and quality, but did not adequately consider the subjective acceptance of AI-generated documents by doctors and patients. Understanding users' attitudes and concerns is crucial for the successful application of AI technology in the medical field. Future research should consider more diverse physician samples, rigorously examine and mitigate biases in training data, explore hybrid approaches that combine the strengths of LLMs and human expertise, and assess user acceptance of AI-generated content. These steps will enhance the credibility and comprehensiveness of research findings and facilitate the effective integration of AI technology in medical practice (30).

## Conclusion

This study provides strong evidence for the efficiency and reliability of Claude 3.5-Sonnet in generating discharge summaries for kidney disease patients. The significant time advantage demonstrated by the AI model, along with accuracy and quality comparable to human physicians, highlights its enormous potential in improving the efficiency of medical document generation. However, successfully integrating AI technology into medical practice still faces many challenges and requires further research and optimization. Future work should focus on expanding the scope of research, optimizing AI model performance, exploring broader application scenarios, and addressing related ethical and privacy issues. Through continuous effort and innovation, AI technology has the potential to become a powerful tool for improving the quality and efficiency of medical services, ultimately benefiting patients and the entire healthcare system.

## Data Availability

The raw data supporting the conclusions of this article will be made available by the authors, without undue reservation.
